# Correction: An overview of atmospheric water harvesting methods, the inevitable path of the future in water supply

**DOI:** 10.1039/d3ra90037a

**Published:** 2023-05-03

**Authors:** Zahra Ahrestani, Sadegh Sadeghzadeh, Hosein Banna Motejadded Emrooz

**Affiliations:** a MSc of Chemistry and Materials Technologie, Institute of Materials Chemistry, Faculty of Chemistry, University of Vienna Vienna Austria; b MSc of NanoTechnology, School of Advanced Technologies, Iran University of Science and Technology Tehran Iran; c School of Advanced Technologies, Iran University of Science and Technology Tehran Iran sadeghzadeh@iust.ac.ir; d School of Advanced Technologies, Iran University of Science and Technology Tehran Iran

## Abstract

Correction for ‘An overview of atmospheric water harvesting methods, the inevitable path of the future in water supply’ by Zahra Ahrestani *et al.*, *RSC Adv.*, 2023, **13**, 10273–10307, https://doi.org/10.1039/D2RA07733G.

The authors regret that incorrect versions of Fig. 8, 10, 11, 13, 14, 20, 21 and 29 were included in the original article. The correct versions of Fig. 8, 10, 11, 13, 14, 20, 21 and 29 are presented below.

**Fig. 1:**
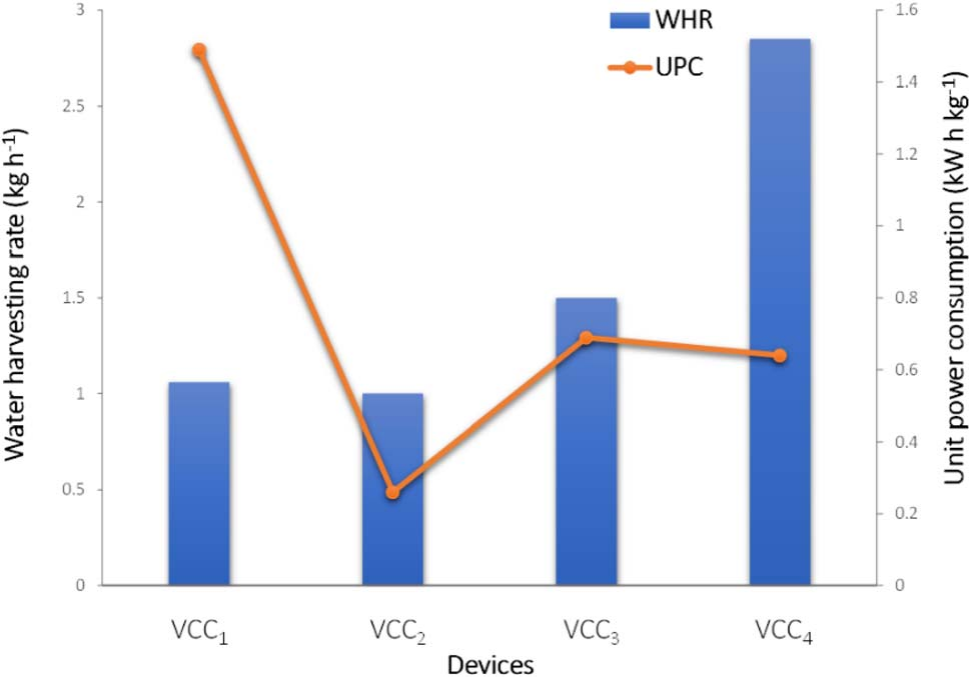
Unit power consumption (UPC) and water harvesting rate (WHR) diagram of VCC condensation systems.

**Fig. 2:**
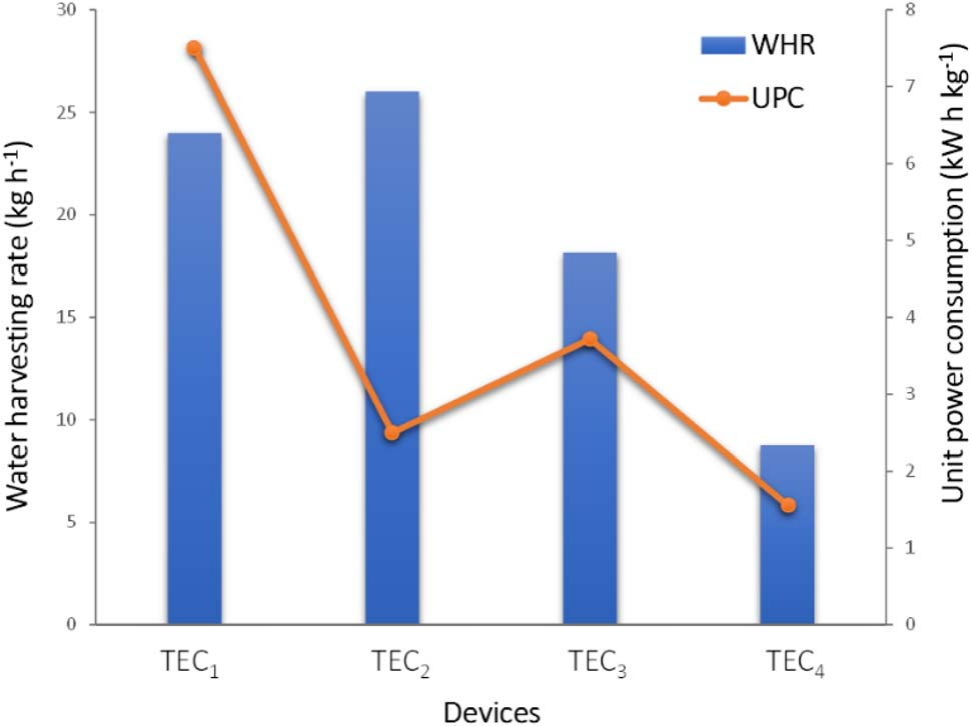
Unit power consumption (UPC) and water harvesting rate (WHR) diagram of TEC condensation systems.

**Fig. 3:**
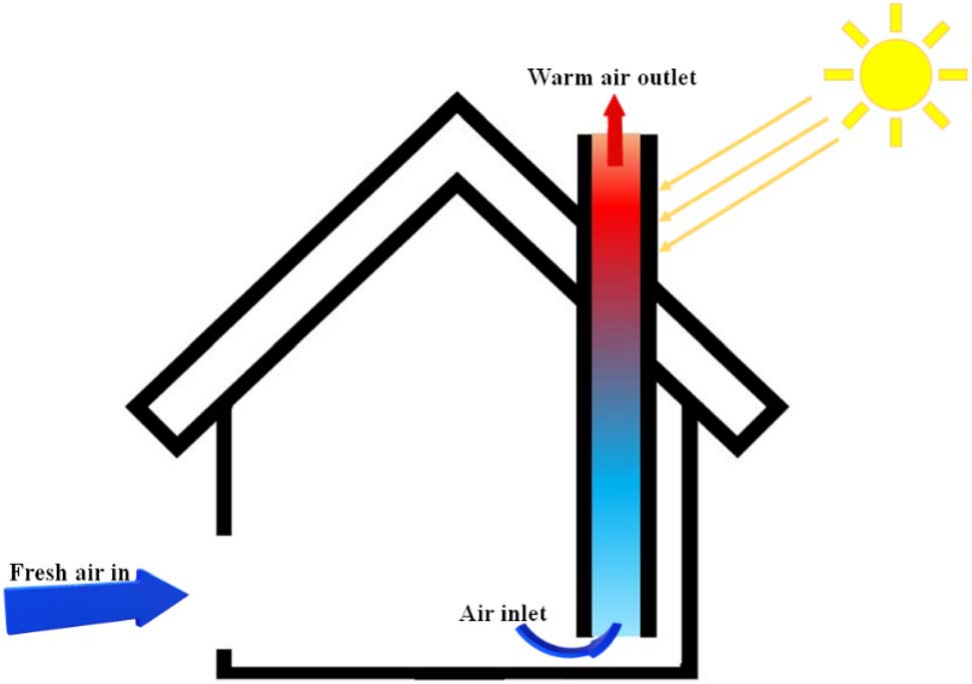
Schematic of solar chimney.

**Fig. 4:**
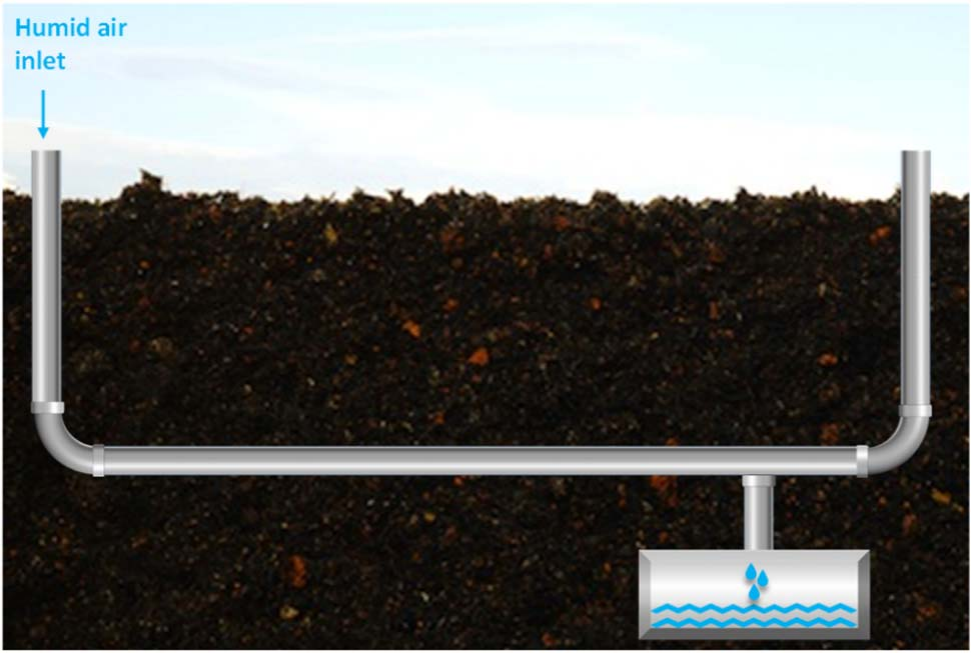
Schematic of condensation drinking water production method.

**Fig. 5:**
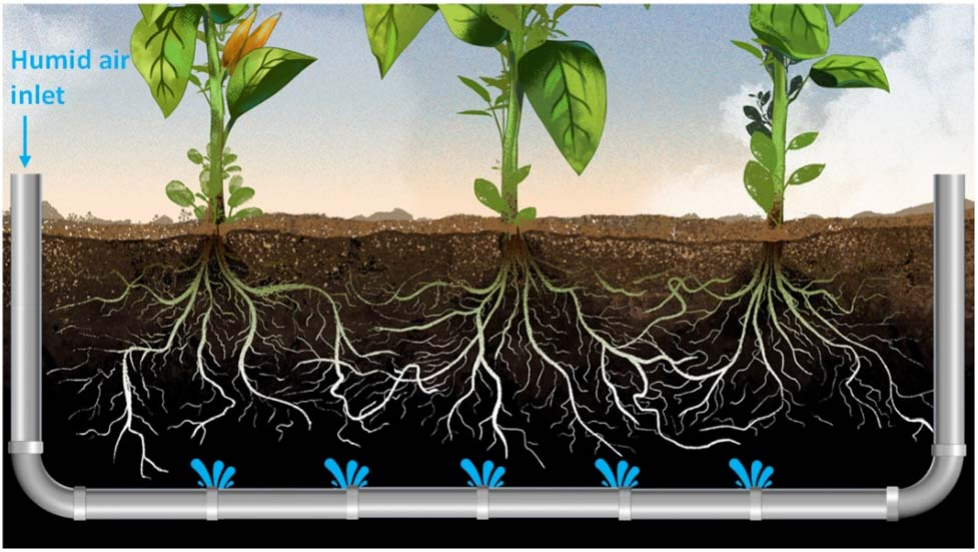
Schematic of condensation water production method for irrigation of agricultural lands.

**Fig. 6:**
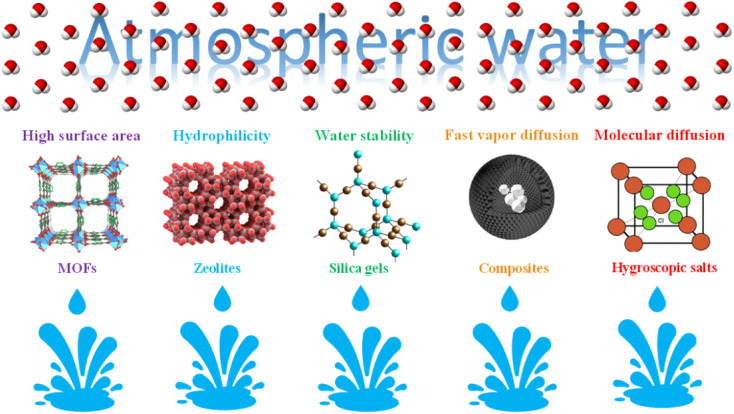
Properties of desiccants in sorption methods AWH.

**Fig. 7:**
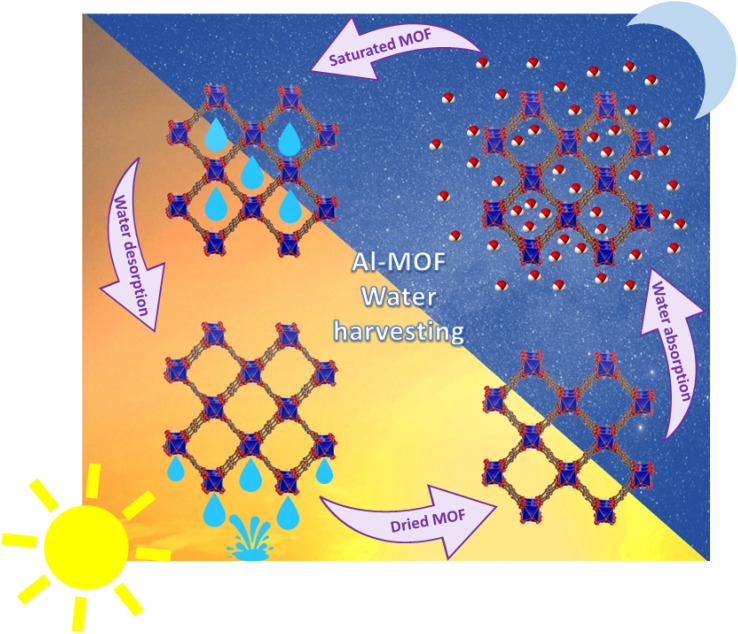
Steps of atmospheric water harvesting used desiccants.

**Fig. 8:**
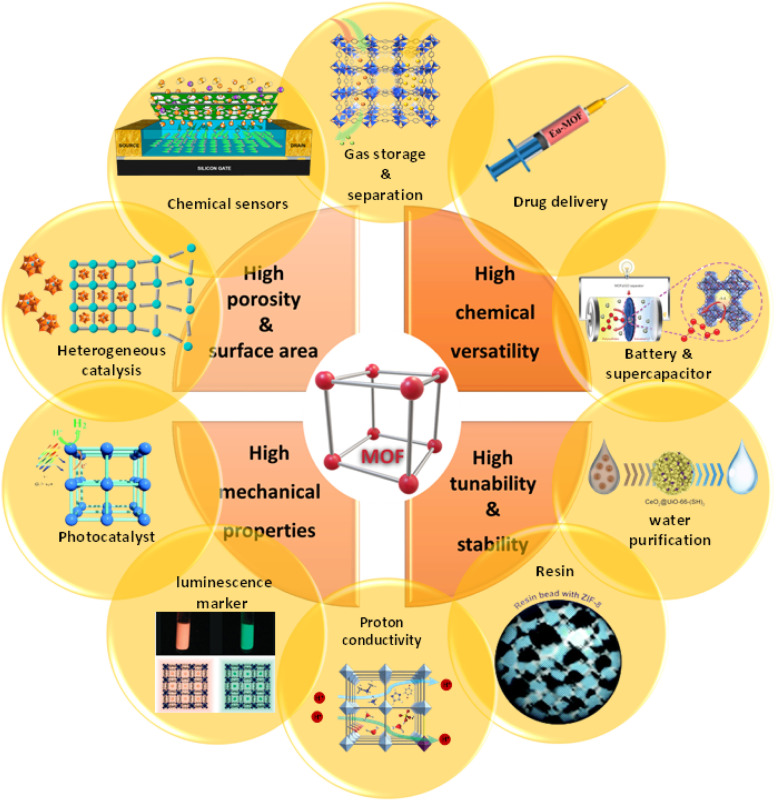
Different properties and applications of MOFs: gas storage and separation. Reproduced from ref. 119 with permission from *Materials Today*. Drug delivery. Reproduced from ref. 120 with permission from *Inorganic Chemistry*. Battery and supercapacitor. Reproduced from ref. 121 with permission from *Nature Energy*. Water purification. Reproduced from ref. 122 with permission from *ACS Applied Materials & Interfaces*. Resin. Reproduced from ref. 123 with permission from *Inorganic Chemistry Frontiers*. Proton conductivity. Reproduced from ref. 124 with permission from *Advanced Materials*. Luminescence marker. Reproduced from ref. 125 with permission from *Journal of the American Chemical Society*. Photocatalyst. Reproduced from ref. 126 with permission from *Chemical Communications*. Heterogeneous catalysis. Reproduced from ref. 127 with permission from *ACS Catalysis*. Chemical sensors. Reproduced from ref. 128 with permission from *ACS Applied Materials & Interfaces*.

The Royal Society of Chemistry apologises for these errors and any consequent inconvenience to authors and readers.

## Supplementary Material

